# Existing Digital Health Technology Index Summary Report for Older Adults Living with Neurocognitive Disorders (Mild and Major) and Their Informal Caregivers: An Environmental Scan

**DOI:** 10.3390/geriatrics9040085

**Published:** 2024-06-22

**Authors:** Ambily Jose, Maxime Sasseville, Ellen Gorus, Anik Giguère, Anne Bourbonnais, Samira Abbasgholizadeh Rahimi, Clémence Balley, Ronald Buyl, Marie-Pierre Gagnon

**Affiliations:** 1 VITAM Research Center in Sustainable Health, Quebec City, QC G1J 2G1, Canada; ambily.baburaj.1@ulaval.ca (A.J.); maxime.sasseville@fsi.ulaval.ca (M.S.); anik.giguere@fmed.ulaval.ca (A.G.); clemence-justine-aichatou.djentre-balley.1@ulaval.ca (C.B.); 2 Faculty of Nursing Sciences, Université Laval, Quebec City, QC G1V 0A6, Canada; 3 Department of Gerontology, Frailty in Ageing Research Group, Vrije Universiteit Brussel, 1090 Brussels, Belgium; ellen.gorus@uzbrussel.be; 4 Department of Family and Emergency Medicine, Faculty of Medicine, Université Laval, Quebec City, QC G1V 0A6, Canada; 5 Faculty of Nursing, Université de Montréal, Montreal, QC H3T 1J4, Canada; anne.bourbonnais@umontreal.ca; 6 Department of Family Medicine, McGill University, Montreal, QC H3A 0G4, Canada; samira.rahimi@mcgill.ca; 7 Mila-Quebec AI Institute, Montreal, QC H2S 3H1, Canada; 8 Department of Biostatistics and Medical Informatics, Faculty of Medicine and Pharmacy, Vrije Universiteit Brussel, 1090 Brussels, Belgium; ronald.buyl@vub.be

**Keywords:** digital health, aging, cognitive impairment, dementia, environmental scan, e-health, assisted living, assistive technology, cognitive assistance

## Abstract

Digital health has added numerous promising solutions to enhance the health and wellness of people with neurocognitive disorders (NCDs) and their informal caregivers. (1) Background: It is important to obtain a comprehensive view of currently available technologies, their outcomes, and conditions of success to inform recommendations regarding digital health solutions for people with NCDs and their caregivers. This environmental scan was performed to identify the features of existing digital health solutions relevant to the targeted population. This work reviews currently available digital health solutions and their related characteristics to develop a decision support tool for older adults living with mild or major neurocognitive disorders and their informal caregivers. This knowledge will aid the development of a decision support tool to assist older adults and their informal caregivers in their search for adequate digital health solutions according to their needs and preferences based on trustable information. (2) Methods: We conducted an environmental scan to identify digital health solutions from a systematic review and targeted searches in the grey literature covering the regions of Canada and Europe. Technological tools were scanned based on a preformatted extraction grid. We assessed their relevance based on selected attributes and summarized the findings. (3) Results: We identified 100 available digital health solutions. The majority (56%) were not specific to NCDs. Only 28% provided scientific evidence of their effectiveness. Remote patient care, movement tracking, and cognitive exercises were the most common purposes of digital health solutions. Most solutions were presented as decision aid tools, pill dispensers, apps, web, or a combination of these platforms. (4) Conclusions: This environmental scan allowed for identifying current digital health solutions for older adults with mild or major neurocognitive disorders and their informal caregivers. Findings from the environmental scan highlight the need for additional approaches to strengthen digital health interventions for the well-being of older adults with mild and major NCDs and their informal and formal healthcare providers.

## 1. Introduction

It is anticipated that the number of people with mild or major neurocognitive disorders (NCDs) will rise to 70 million by 2030 and 139 million by 2050 [1]. Based on the DSM-5-TR, we refer to mild cognitive impairment as “mild neurocognitive disorders” and dementia as “major neurocognitive disorders” in this paper [2]. NCDs raise complex challenges for older adults, their families, and society since they lead to progressive decline in cognitive functioning and activities of daily living, resulting in people becoming more dependent on the support of others, social exclusion, caregiver stress, and increasing care costs [3]. NCDs negatively affect the quality of life of older adults as they suffer problems with memory, thinking, orientation, language, comprehension, and judgment [4].

Most older adults prefer to live in their own homes and want to maintain their independence and autonomy for as long as possible [5]. Digital health solutions can improve the independence and autonomy of people living with NCDs and provide knowledge and assistance to their caregivers [6,7,8]. Hence, these assistive devices can help to reduce reliance on other people and care systems and help them stay in their homes longer. Thus, digital health is considered a promising solution for these people [9].

Many digital health initiatives have been developed in recent years to improve the quality of life for older adults with NCDs [10,11]. Digital health technology is the umbrella term used to describe devices or systems that “increase, maintain or improve capabilities of individuals with cognitive, physical or communication disabilities” [12]. The majority of these are behaviorally based health interventions that are provided via the Internet, mobile devices, electronic/digital communication processes, and related technologies to close gaps in an individual’s capacity to lead a complete, self-sufficient, and happy life [13].

To help guide recommendations regarding digital health solutions for people with NCDs and their caregivers, it is critical to obtain a thorough understanding of the technologies that are currently on the market and to record their results and success conditions. To find current digital health solutions that are pertinent for individuals with mild or major NCDs, as well as their family members and unpaid caregivers in Canada and Europe, we conducted an environmental scan.

This study was funded by the Fonds Wetenschappelijk Onderzoek (FWO) and the Fonds de Recherche du Québec (FRQ) bilateral agreement [grant number G0D8518N]. This study was conducted as part of a collaborative project involving partners from Flanders (Belgium) and Quebec (Canada). This project aims to identify digital health solutions for older adults living with mild or major NCDs and their informal caregivers and to document their characteristics to inform the implementation of such solutions in Europe and Canada. The specific objectives are as follows:

1. Inventory digital health solutions for the targeted populations available from public sources in Europe or Canada.

2. Summarize the characteristics of these identified digital health solutions, including their features and outcomes, implementation factors, and conditions of success.

## 2. Methods

To accomplish these goals, we ran an environmental scan as this approach is regarded as a useful data gathering and assessment tool for planning and decision-making, policy exploration, analysis of complex issues, and evaluation of recent evidence. There is no gold standard for the environmental scan method despite its growing popularity in health research as a methodological approach to investigating a particular health issue [14]. An environmental scan involves seeking, gathering, and interpreting information to identify knowledge gaps and orient future action [15]. Several studies have described the usefulness of environmental scans for assessing community needs for program and policy development [16,17,18]. Our environmental scan followed the general steps of a literature review, i.e., identification, selection, extraction, synthesis, and interpretation. We did not assess the scientific quality of the publications included, as this is not the goal of an environmental scan.

### 2.1. Search Strategy

We performed comprehensive bibliographic and grey literature searches in scientific databases and public websites from various organizations and governments to identify digital health solutions for older people with mild or major NCDs or dementia and their informal caregivers. Initial data searches were performed through a systematic review by Dequanter et al. [8], covering the period from 2013 to 2018. Initial data were updated until June 2023 by two research assistants by gathering all available digital health solutions in their respective jurisdictions (Canada and Europe) through general and specific web searches (see Supplementary Materials). Finally, all identified solutions were reviewed by experienced investigators (MPG, MS, RB).

### 2.2. Inclusion and Exclusion Criteria

We included all digital health solutions whose main purpose is supporting and improving health and well-being in the daily life of people with NCDs or their caregivers, such as apps providing information about health or related services, cognitive exercises and games, virtual assessments, etc. The digital solution could also target other groups but had to be suitable for people with NCDs or their caregivers. We included digital solutions produced within the 2013 to 2023 timeframe and available in Europe or Canada. We focused on these regions because they have mostly public healthcare systems, and we wanted to ensure that digital health solutions could be presented to people with NCDs and their informal caregivers living in these regions. We included relevant solutions provided by any public or private entity, free of charge or those requiring some payment. Although we were interested in documenting scientific evidence about the solutions, we did not exclude those without. Digital health solutions that were not currently available or for which we were unable to confirm availability were excluded. Solutions only available in languages other than English, French, or Dutch were also excluded since these solutions could not be easily transferred to the context of our project.

### 2.3. Data Extraction

We used an extraction grid to document the characteristics of digital health solutions. The collected information included the solution’s name, purpose, software used, target population, domain, features, geographic availability, summary of the invention, scientific evidence of impacts and evidence details, and primary author or company contact information (email and phone number). When this information was available, we also documented the key attributes of digital health solutions identified through a qualitative project of this team [19]. These attributes were digital health literacy, cognitive and physical limitations, ease of use, design, compatibility, affordability, and relevance.

We piloted the extraction grid on a sample of five solutions. Then, one author (AJ) extracted all identified technologies, and another author (MS or MPG) checked for accuracy.

### 2.4. Data Analysis

We gathered detailed information based on the selected attributes and listed all available digital health solutions for the targeted populations in Europe and Canada. This provided adequate data for the first objective. Then, we described each technology based on the selected attributes, narratively synthesized quantitative and qualitative data, and triangulated the results to understand implementation factors and conditions of success.

## 3. Results

We used a narrative approach with charts and figures to summarize the results according to the key characteristics of the technological solutions. Classifying digital health solutions based on their main function reveals their importance for patients and informal caregivers and their documented advantages, implications, and potential drawbacks.

### 3.1. Geographical Availability

**Figure 1:**
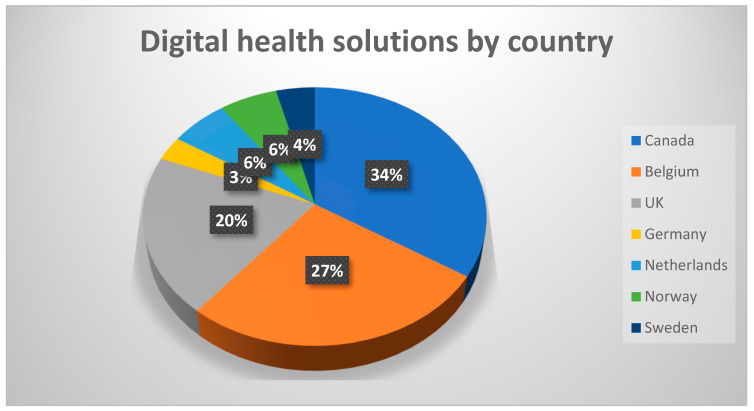
Distribution of the countries where digital health solutions are available.

From the various sources consulted, we identified 100 distinct digital solutions available in Canada, Belgium, the UK, Germany, the Netherlands, Norway, and Sweden (Figure 1).

### 3.2. Target Population

Most digital health solutions target older adults in general (56%), while 17% of the solutions are specifically for older people with NCDs. However, 23% of solutions apply to all age groups.

### 3.3. Available Evidence

We considered scientific evidence supporting digital health interventions and found that most solutions do not have any evidence of their effectiveness. Only 28 of the 100 solutions identified present scientific evidence of their results, most often based on clinical trials. Other types of evidence, such as media releases, awards and recognitions, user reviews, and feedback, are available for 34 solutions. However, no supporting scientific or non-scientific evidence is reported for the remaining 38 solutions.

### 3.4. Purpose

**Figure 2:**
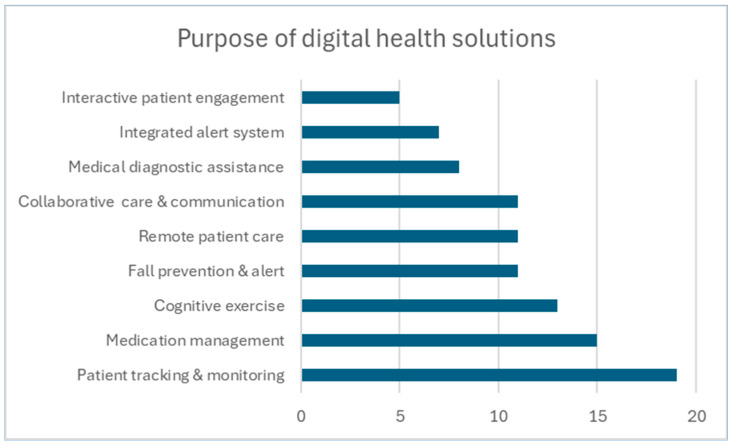
Purpose of digital health solutions.

As seen in Figure 2, classification based on their main purpose shows that most digital health solutions are for remote patient care (23), focus on cognitive exercise (20), or track movements (17). Twelve solutions aim at fall prevention, and ten offer alert and security functions. The other types of solutions offer decision support (2) and robotic interactions (2).

### 3.5. Technological Support

This parameter defines the technology platforms on which the solutions are based. There are six main categories of platforms (Table 1) that are briefly described below.

#### 3.5.1. Aid Tool

Identified various healthcare aid tools that support mobility and decision-making. It uses wearable technology solutions and walker sensors for neuromuscular rehabilitation for patients with brain injury, neurological disorders, or mobility challenges. These solutions are user-friendly, affordable, and relevant for people with dementia.

#### 3.5.2. Aid Tool and App

This category is an extension of the former and includes the aid tool and app. These are useful for older adults who require personalized rehabilitation plans. They help in early complication identification by using an app that is useful for daily remote medical care or exercise videos with a system to provide feedback or reinforcement.

#### 3.5.3. App

This category includes mobile systems that help to ensure client safety, for instance, by keeping track of the person’s movements. An example is an electronic door opener that is activated by a key app. These solutions are generally easy to use, safe, and acceptable as the client determines who receives access to their information.

#### 3.5.4. Pill Dispenser and App

Electronic pill dispenser technology helps people with cognitive problems who require regular medicine by providing helpful reminders through chimes and lights and phone or text alerts to remind the person of any delay in taking their medication. This is facilitated by the coordination with pharmacy and care teams by pre-set pill bottles to alert and message medication schedules. It also allows integration with healthcare professionals and pharmacies for real-time tracking and updates. No technical skills are required for easy usage. However, this solution is specific to pills and will not work with injections, drops, inhalers, etc. It is available only through partner pharmacies.

#### 3.5.5. Web

Remote patient monitoring can be performed by using secure web tools. For these types of solutions, a case manager often coordinates the care plan and ensures the proper functioning of the network. Supporting functions for patients and their relatives are coordinated through a web portal for primary care coordination and support services for palliative care.

#### 3.5.6. Web and App

Adding mobile apps to the previous category allows for extended coverage and functionalities. Tracking is facilitated, and coordination of care is improved. These solutions are notably useful to support the mobility of people with cognitive problems.

Table 2 provides examples of specific technologies that are available for each category of platforms.

## 4. Discussion

Digital health technology utilization has changed the way older adults live in and out of their homes. Virtual assistants empower people living with NCDs to live independently at home for longer. Technology-based education has improved the knowledge, skills, and attitudes of the people who are dealing with them by supporting their cognitive functions, activities of daily living, and safety [20]. Based on the individual requirements, the selected solutions may differ from each other in terms of objectives, usage mode, or characteristics, even if they were used for the same purposes. Therefore, a detailed assessment of the features and context of the use of solutions is essential for selecting the one that best suits each user [21]. Similarly, this study also intends to elicit more information on the available technologies to develop a decision support tool to meet individual user requirements.

As cognitive impairment progresses, caregiver support is vital [7,18,22]. Hence, technologies that support decreasing caregivers’ burden, such as support apps and telemonitoring systems, are useful. Older adults will need highly effective forms of support from the point of diagnosis to ensure that they remain connected to their family and community and can live well with this long-term illness. The results of a multimedia system intervention at a residential home showed that all residents were able to use the system with a high degree of autonomy via its adaptable interfaces. Family members and caregivers reported high levels of enjoyment when observing the residents using the systems. Accessing favorite music and photographs reportedly caused great delight in many residents, and depression and anxiety symptoms decreased [23].

Many NCD-focused publications showcase a range of innovative designs and creative interventions that have been developed for the health promotion and supportive care of older adults with NCDs [3,12,20,24,25,26]. Innovative, promising solutions address the twin impacts of an aging population with increased cognitive impairment and the shortage of caregivers [6,27]. Such occasions can be effectively dealt with through the utilization of digital health solutions; robots; artificial intelligence-driven sensors; embedded and wearable devices, including video; and other monitoring systems integrated into the home environment, which can offer increased security, safety, health management, and social systems to support both the older adult with NCDs as well as informal and formal caregivers [12,23,28]. Some studies strongly support that everyday interactive products have the potential to support older adults to live well with NCDs by improving their quality of life by preserving a person’s sense of self; reducing anxiety and agitation; and bringing comfort, joy, and pleasure [29]. We were able to identify solutions from the above categories.

Although digital health technologies offer many benefits, they can challenge and trouble older adults with cognitive disorders [29]. Older adults may be able to incorporate technologies during the onset of symptoms, but later, they can experience challenges with them. There are many challenges in introducing new technologies to older adults [3,12,23,28]. Technologies can be intrusive and, if used without the consent of the cognitively impaired person, deprive them of their rights. These challenges can be managed by developing specific frameworks, guidelines, and regulations for technology adoption among caregivers and people with NCDs [23,28]. Functionalities that may prove advantages in the early stages of their condition may result in a risk of anxiety or distress. Significantly, attention should be paid when a technology or device is no longer desirable for a person whose NCD may have progressed [9,24,28]. In our review, we found that only 28% of the identified digital health solutions had scientific evidence supporting their effectiveness. This limited evidence base raises concerns about the overall quality and reliability of these solutions. Given these limitations, we recommend that future research prioritize the generation of high-quality scientific evidence through well-designed studies, including randomized controlled trials with larger sample sizes and longer follow-up periods. Furthermore, there is a need for standardized reporting guidelines to ensure consistency and transparency in evaluating digital health interventions. Some technologies do not exactly meet their needs, although they claim to be effective. In fact, no study included in our systematic review [8] clearly analyzed how the technology is used by people with NCDs in their daily activities. More studies are required to monitor usage, usability, effectiveness, preferences, and barriers related to the uptake of these solutions by people with dementia or other NCDs and their informal caregivers. Therefore, the development of a decision support tool is highly recommended to meet individual requirements based on each one’s capabilities. Our review focuses on digital health solutions available in Canada and Europe, which limits the generalizability of our findings to other regions. This geographical scope was chosen because these areas have predominantly public healthcare systems, allowing for a more homogeneous comparison of digital health interventions. However, digital health solutions available in other parts of the world, particularly in regions with different healthcare models and technological infrastructures, may present different outcomes and implementation challenges. Future research should aim to include a more diverse range of geographical contexts to provide a comprehensive global perspective on the implementation and effectiveness of digital health solutions for older adults with neurocognitive disorders.

Based on the available technologies reviewed in this study, pill dispensers and apps remind older adults of NCDs to take their medications on time, cognitive exercises claim to limit their memory problems, and mobile applications may assist them in activities like cooking or self-management skills. Other digital health solutions offer safety and social support by connecting with family members and caregivers. Interactive devices like talking puppies, dolls, or robots may offer time orientation, stimulate alertness, enhance communication, provide entertainment, and alleviate feelings of loneliness.

## 5. Conclusions

This environmental scan allowed for identifying current digital health solutions for older adults with mild or major neurocognitive disorders and their informal caregivers. Only 28 of the 100 solutions identified present scientific evidence of their results. Based on the features of these solutions, it seems possible to design an effective decision-making tool that could support them when deciding to use such technology. Findings from the environmental scan highlight the need for additional approaches to strengthen digital health interventions for the well-being of older adults with mild and major NCDs and their informal and formal healthcare providers. Technology development for people living with major and minor NCDs requires a keen assessment based on each person’s requirements. When an appropriate intervention is used in a timely manner, it could lead to a greater feeling of independence and confidence among older adults with NCDs.

## Figures and Tables

**Table 1:** Types of platforms and the number of solutions identified.

Type of Platform Used	Number of Solutions
Aid tool	26
Aid tool + App	10
App	21
Pill dispenser + App	14
Web	8
Web + App	17
Others	4

**Table 2:** Types of platform categories and examples of solutions identified.

Category	Examples
Wearable	Smartwatches, necklaces, or bracelets with GPS, falls monitoring, and medical monitoring of pulse respiration, oxygen levels, etc.
Handheld devices	Tablets with multimedia technology, Personal Digital Assistant (PDA), Global Positioning Systems (GPS) trackers, fitness trackers, smartphones, etc.
Mobility aids	Smart canes or smart wheelchairs with built-in sensors
Voice-activated assistants	Siri, Alexa/Echo, HUG
Distributed systems	Smart homes with integrated sensor systems for light, heat, and window coverings
Situation specific robots	Soothing and emotional response enhancer robots like PARO

## Supplementary Materials

The following supporting information can be downloaded at: https://www.mdpi.com/article/10.3390/geriatrics9040085/s1, Table S1: Search Strategy and Data Sources.
