# Supplementary material for: Existing Digital Health Technology Index Summary Report for Older Adults Living with Neurocognitive Disorders (Mild and Major) and Their Informal Caregivers: An Environmental Scan
Source: Geriatrics (Basel). 2024 Jun 22;9(4):85. doi: 10.3390/geriatrics9040085 (PMC11270236; doi:10.3390/geriatrics9040085)
Supplement: Supplementary file 1 [file geriatrics-09-00085-s001.zip › geriatrics-2986889-supplementary.pdf]

Table S1

Search Strategy and Data Sources

Search strategy

Terms dealing with targeted population	Medical Subject Heading (MeSH) terms	Terms dealing with eHealth solutions	Additional terms
elderly, older people, caregiver, family caregiver, family caregiving, informal care, informal care givers	mild cognitive impairment, mild neurocognitive disorder, dementia, Alzheimer' Disease, neurodegenerative disorder, neurocognitive disorder, memory support, memory assist, memory help, cognition support	ICT services, information and communication technology, e-Health, medical informatics, health informatics, mobile health, mhealth, telemedicine, telehealth, telecare, mobile devices, mobile applications, self-help applications, self-help devices, self-management applications, handheld computers, tablets, mobile phones, smartphones, personal digital assistant, mobile technology, healthcare robotics, assistive technology intelligent systems, networked technology, telemonitoring, ambient assisted living, active and assisted living, e-learning, ADL technologies, virtual reality.	prevention, prevent, adoption, use, non-use, acceptance, community-dwelling, community-based, nursing homes, day care centers, gerontechnology, psychoeducation prescription

Data sources

General search via Google website, social networks (e.g., Twitter, Instagram), clinical and public health organizations (e.g., Health academia, WHO…)

Targeted searches

Types of organizations	Organizations
Government agencies	Canadian Frailty Network, Public Health Agency of Canada, AgeWell, European Commission projects funded through the EU, Canadian Consortium on Neurodegeneration in Aging
Public and private organizations	Digital Alzheimer Center of the Vrije Universiteit, Medical Center Amsterdam, Cordis europa, Digital single market, Canadian Institutes of Health Research, Active and Assistive Living programs, H2020 programs, Alzheimer Europe<br>Advocacy organizations patients/users (e.g. Dementia Alliance International)<br>Research centers (e.g. Centre for aging Brain innovations)<br>Industries
Practitioner and public health-related organizations	Rural Dementia Action research, Canadian Medical Association
Patient and caregiver associations	Alzheimer Society Canada, Alzheimer disease international, Women's brain health initiative
Other relevant sites	Canadian Geriatrics Society, Canadian Academy of Geriatric Psychiatry TheGerontechnologist.com
